# Getting stuck with pornography? Overuse or neglect of cybersex cues in a multitasking situation is related to symptoms of cybersex addiction

**DOI:** 10.1556/JBA.4.2015.1.5

**Published:** 2015-03-18

**Authors:** JOHANNES SCHIEBENER, CHRISTIAN LAIER, MATTHIAS BRAND

**Affiliations:** ^1^Department of General Psychology: Cognition, University of Duisburg-EssenDuisburgGermany; ^1^Department of General Psychology: Cognition, University of Duisburg-EssenDuisburgGermany; ^1^Department of General Psychology: Cognition, University of Duisburg-EssenDuisburgGermany; ^2^Erwin L. Hahn Institute for Magnetic Resonance ImagingEssenGermany; ^1^Department of General Psychology: Cognition, University of Duisburg-EssenDuisburgGermany; ^2^Erwin L. Hahn Institute for Magnetic Resonance ImagingEssenGermany

**Keywords:** Internet addiction, cybersex, Internet pornography, multitasking, cue-reactivity, psychopathological symptoms

## Abstract

**Background and aims:**

Some individuals consume cybersex contents, such as pornographic material, in an addictive manner, which leads to severe negative consequences in private life or work. One mechanism leading to negative consequences may be reduced executive control over cognition and behavior that may be necessary to realize goal-oriented switching between cybersex use and other tasks and obligations of life.

**Methods:**

To address this aspect, we investigated 104 male participants with an executive multitasking paradigm with two sets: One set consisted of pictures of persons, the other set consisted of pornographic pictures. In both sets the pictures had to be classified according to certain criteria. The explicit goal was to work on all classification tasks to equal amounts, by switching between the sets and classification tasks in a balanced manner.

**Results:**

We found that less balanced performance in this multitasking paradigm was associated with a higher tendency towards cybersex addiction. Persons with this tendency often either overused or neglected working on the pornographic pictures.

**Discussion:**

The results indicate that reduced executive control over multitasking performance, when being confronted with pornographic material, may contribute to dysfunctional behaviors and negative consequences resulting from cybersex addiction. However, individuals with tendencies towards cybersex addiction seem to have either an inclination to avoid or to approach the pornographic material, as discussed in motivational models of addiction.

## INTRODUCTION

Most people use the Internet in a functional way. One characteristic of functional, non-problematic Internet use is that the Internet can be applied to achieve and fulfill needs and goals (Brand, Young & Laier, 2014). It has been argued that functional Internet users can interrupt Internet sessions when other obligations urge or that they can easily end Internet use when the goals are reached. In other words, functional Internet users are able to switch between the Internet and other activities in a goal-adequate way. However, in the last years a phenomenon emerged which is often called Internet addiction. The phenomenon has not yet been incorporated into international classification systems (ICD-10; DSM-IV-TR; DSM-V; Dilling, Mombour & Schmidt, 1999; Saß, Wittchen & Zaudig, 1996), but Internet Gaming Disorder has been included in the appendix of the DSM-V. Although the classification as behavioral addiction is still discussed (cf., Brand et al., 2014; Charlton & Danforth, 2007; Davis, 2001; Kuss & Griffiths, 2012b; Kuss, Griffiths, Karila & Billieux, 2013; LaRose, Lin & Eastin, 2003; Meerkerk, van den Eijnden, Vermulst & Garretsen, 2009; O’Brian, 2010; Petry & O’Brien, 2013; Starcevic, 2013; Young, 2004), many authors argue that the symptoms are comparable to those of addictions: Affected individuals feel a strong urge to consume Internet content, have reduced control over their Internet use, make unsuccessful attempts to reduce Internet consumption, show symptoms of withdrawal when being offline, neglect social and professional activities, and continue Internet use despite repeated negative consequences (e.g., Griffiths, 2000; Morahan-Martin, 2008; Weinstein & Lejoyeux, 2010; Young, 1998).

A key feature of Internet addiction is seen in loss of control over consumption (Brand et al., 2014). The current study aims at better understanding the mechanisms behind loss of control. We suggest that one of these mechanisms is a failure to exert cognitive control over cognition and behavior that is necessary to switch between the Internet and other tasks of life in a goal-adequate way. Here, we concentrate on cybersex addiction – a specific type of Internet addiction (see e.g., Davis, 2001; Kuss & Griffiths, 2012a; Meerkerk, van den Eijnden & Garretsen, 2006). A recent theoretical approach towards explaining Internet addiction was suggested by Brand et al. (2014). Based on the cognitive-behavioral model of pathological Internet use by Davis (2001), Brand et al. (2014) suggested three models describing predictors and mechanisms of functional Internet use, generalized Internet addiction, and specified Internet addiction, respectively. Cybersex addiction is one main type of a specific Internet addiction (Meerkerk et al., 2006; Young, 2008), besides Internet Gaming. Brand et al. (2014) propose that two main person characteristics render an individual vulnerable for the development and maintenance of a specific Internet addiction, such as cybersex addiction. The first person characteristic is a non-specific predisposition with psychological-psychiatric symptoms. Several studies indeed showed that tendencies towards cybersex addiction are correlated with obsessive-compulsive symptoms, depression, psychoticism, anxiety, loneliness, or general psychological well-being (e.g., Brand et al., 2011; Kuss & Griffiths, 2012a; Pawlikowski & Brand, 2011; Pawlikowski, Nader, et al., 2013; Philaretou, Mahfouz & Allen, 2005; Putnam, 2000; Schwartz & Southern, 2000). The second person characteristic is a specific predisposition for receiving high gratification from specific content. For example, studies found that an individual may be predisposed for cybersex addiction by a high sexual arousability (Bancroft & Vukadinovic, 2004; Cooper, Delmonico & Burg, 2000; Cooper, McLoughlin & Campell, 2000; Kafka, 2010; Salisbury, 2008). Repeated positive reinforcement (e.g., due to sexual arousal) and negative reinforcement (e.g., due to reduction of negative emotions) are suggested to lead to conditioning and therefore to repeated and increasing Internet use, despite negative consequences (Brand et al., 2014). Furthermore, individuals can become conditioned to immediately react to addiction related cues by experiencing cue-reactivity (= immediate cue-induced experience of arousal) and craving (= strong urge to consume cybersex material). This idea has been supported with regard to cybersex in previous studies (Brand et al., 2011; Laier, Pawlikowski, Pekal, Schulte & Brand, 2013).

Brand et al. (2014) argued that loss of control over consumption is a main mechanism in Internet addiction. Being conditioned towards Internet use “makes it increasingly harder for an individual to cognitively control the Internet use, even though negative consequences related to the Internet overuse are experienced in the long run” (p. 3; Brand et al., 2014). Brand et al. (2014) suggested that cognitive control is particularly reduced when individuals are confronted with their addiction-specific material (e.g., pornographic material).

In general, the implementation of control over behavior and thought is a cognitive capacity implemented by a set of executive control functions (Anderson, Anderson & Jacobs, 2008; Cools & D’Esposito, 2011) guided particularly by the prefrontal cortex (e.g., the dorsolateral part) and some sub-cortical regions (e.g., regions in the basal ganglia) (see e.g., Alvarez & Emory, 2006; Jurado & Rosselli, 2007; Stuss & Knight, 2013). Executive control functions are for example attention, inhibition, set-shifting, planning, monitoring, strategy control, and also working memory and decision making (Baddeley, 2003; Borkowsky & Burke, 1996; Jurado & Rosselli, 2007; Miyake et al., 2000; Shallice & Burgess, 1996; Smith & Jonides, 1999).

Pornographic material reduces performance in executive control tasks requiring visual performance or quick reactions (i.e. attention/inhibition) (Macapagal, Janssen, Fridberg, Finn & Heiman, 2011; Most, Smith, Cooter, Levy & Zald, 2007; Prause, Janssen & Hetrick, 2008; Wright & Adams, 1999), working memory (Laier, Schulte & Brand, 2013), or decision making (Laier, Pawlikowski & Brand, 2014). Reduced performance in attention/inhibition and working memory tasks have been found to be associated with higher sexual arousability (Macapagal et al., 2011) or the individual need to masturbate (Laier, Schulte et al., 2013). These findings converge on the view that cognitive control and executive functions can be interfered by processing sexual stimuli.

One domain requiring executive control is goal-oriented multitasking. For example, a cybersex user may be occupied with surfing on pornography websites while at the same time other tasks of life are pondering which cannot be performed in parallel, but only after the consumption of cybersex has been ended. Being able to serially work on the tasks in a goal-oriented, and functional way may involve several aspects of executive control, such as monitoring the completion statuses of different tasks, disengaging from pornographic material, and shifting to other tasks (see e.g., Burgess, 2000; Burgess, Veitch, de Lacy Costello & Shallice, 2000; Manly, Hawkins, Evans, Woldt & Robertson, 2002; Shallice & Burgess, 1996).

Given that multitasking requires executive control processes and given that sexual pictures and addiction-specific material can interfere with executive control, we hypothesized that a reduction in the ability to perform multitasking in environments involving sexual stimuli is a correlate of cybersex addiction. We expected that users with higher tendency towards cybersex addiction “get stuck” with sexual stimuli despite the explicit goal to care for other tasks to the same amount.

Furthermore, given the important role of a psychopathological predisposition for cybersex addiction, we hypothesized that persons who have severer psychopathological problems combined with a weaker ability to control multitasking with pornographic stimuli should suffer from more symptoms of cybersex addiction.

## METHOD

### Participants

We investigated 104 heterosexual males – recruited by local advertisement – at the Department of General Psychology: Cognition at the University of Duisburg-Essen. Advertisement explicated that the study is about Internet pornography use and that legal pornographic material will be presented. Participants received € 10/hour or credits for courses. Table 1 shows sociodemographic characteristics of the sample.

### Measures

Multitasking – Balanced Switching Task porn (BSTporn)

For the current study, the BST – a computerized multitasking paradigm with numbers and shapes, developed earlier by ourselves as a measure of monitoring (Schiebener et al., 2014; Gathmann, Schiebener, Wolf & Brand, 2015) – was equipped with pictures.

**Table 1.** Sociodemographic characteristics of the sample (all: heterosexual males)

	Range	M (SD)
Age (years)	18–50	24.29 (3.96)
Cybersex use (number of cybersex related online sessions per week)^1^	0–7	2.90 (1.84)
Years of school education	9–13	12.57 (0.94)

Breiner	N	

In partnership (yes/no)	50/54	
Children (yes/no)	1/103	

^1^ “How often do you visit sex sites on the Internet”, on one day in two weeks or less (= 0), on one day per week (= 1), on two days per week (= 2), on three days per week (= 3), on four days per week (= 4), on five days per week (= 5), on six days per week (= 6), on seven days per week (= 7).		

In the BSTporn, participants have the aim to proceed to equal amounts on each of four tasks by switching between them. There are two sets of stimuli:

“Person pictures”: Pictures of a man and a woman taking a walk or jogging plus a right- or left-oriented diagonal hatching with thin black lines on the pictures.

“Pornographic pictures”: Contains typical heterosexual pornographic pictures showing vaginal intercourse or oral sex between a man and a woman, taking place either in a room or outdoors.

The four tasks are:

Task 1 (person pictures): Indicate whether the hatching is going to the upper left (press “d”) or right (“f”).

Task 2 (person pictures): Indicate whether the two persons are taking a walk (“j”) or jogging (“k”).

Task 3 (pornographic pictures): Indicate whether the scene is taking place indoors (“d”) or outdoors (“f”).

Task 4 (pornographic pictures): Indicate whether the picture shows vaginal (“j”) or oral (“k”) sex.

With the space bar participants can switch between the two sets. Within a set, the participants can switch between the tasks, by switching between the response keys (“d”, “f”/“j”, “k”). Only one stimulus is presented at a time. Only one of the four tasks has to be performed with each stimulus.

The participants are given three aims: Work on all tasks as equally often as possible, classify the stimuli as correctly as possible, and work on as many stimuli as possible (by making quick responses). They are informed that switching between the sets with the space bar costs time. This rule was used to increase the time participants stay within one set which should increase the load on monitoring.

All subtasks and the overall task are practiced. The experimenters made sure that the task was understood. The task is administered for four minutes, two times. After each time feedback about performance regarding the three aims is provided. After the first time the participants are reminded of the four tasks and the assignment of keys. The outcome measures are:

1: %setPersonPictures (= [number of pictures presented in the set with persons / number of pictures presented during the whole task] * 100).

2: %setPornographicPictures (= [number of pictures presented in the set with pornographic pictures / number of pictures presented during the whole task] * 100).

3: Deviation from set balance. The deviation from set balance is used as the main variable for measuring BSTporn performance. This variable indicates how much a person deviated from working on the two sets to perfectly equal amounts. Higher values indicate more deviation from this goal. The formula is derived from the statistical formula for computing the standard deviation of a sample. First, it was computed which percentage of the overall number of presented stimuli was presented within each of the two sets (denoted below by %setPersonPictures and %setPornographicPictures). From this value the optimal value of equal performance (50% in each set) was subtracted. The result was squared. Results were summed and then divided by two. Then the root was taken. The possible results range from 0% to 50%.

*deviation from set balance *= √ [((%setPersonPictures
– 50)2 + (%setPornographicPictures – 50)2) / 2]

4: Deviation direction: The deviation direction describes towards which set of pictures a participant tended to deviate from balance. The variable ranges from –100 to 100. A value of 0 denotes that in both sets an equal number of pictures was worked on. A value of –100 denotes that only person pictures were worked, +100 denotes that only pornographic pictures were worked on. Formular:

Deviation direction = %setPornographicPictures –
%setPersonPictures.

Psychopathological predisposition – Brief Symptom Inventory (BSI)

In the BSI (Boulet & Boss, 1991) participants indicate how strongly they suffered from 53 psychological or physical symptoms within the last seven days (“0 = not at all” to “4 = extremely”). There are 9 symptom dimensions: Excessive-compulsive symptoms, depression, anxiety, phobic anxiety, psychoticism, somatization, hostility, paranoid ideation, interpersonal sensitivity. Measure: As main measure we used the Global Severity Index (BSI-GSI), representing overall severity of psychopathological symptoms.

Symptoms of cybersex addiction – s-IATsex

The s-IATsex is a short-version of the Internet Addiction Test (Pawlikowski, Altstötter-Gleich & Brand, 2013) modified for Internet sex sites. Terms like “online” and “Internet” were replaced by “online sexual activity” and “Internet sex sites” (e.g., “How often do you find that you stay on Internet sex sites longer than you intended?”). The s-IATsex has twelve items and a five-point scale from 1 (= never) to 5 (= very often). The test consists of two subscales: “loss of control/time management” and “craving/social problems”. Measures: We were interested in the general severity of experienced negative consequences from cybersex consumption. Thus, we used the s-IATsex sum score, potentially ranging from 12 to 60, as main measure (Cronbach’s alpha = .84). The s-IATsex has been used in several previous studies and is described there in more detail, for example Laier, Pawlikowski, Pekal et al. (2013).

### Statistical analyses

The data were analyzed with IBM, SPSS Statistics Version 21.0. Correlations are Pearson’s correlations, interactions between two variables as predictors of a single variable were analyzed with hierarchical moderated regression analysis (predictors centralized according to Cohen, Cohen, West & Aiken, 2003).

### Ethics

All participants gave written informed consent prior to the investigation and the study was approved by a local ethics committee.

## RESULTS

On average, the samples s-IATsex score and the BSI-GSI were in the normal range, as known from previous analogue samples (Brand et al., 2011; Laier, Pawlikowski, Pekal et al., 2013). S-IATsex and BSI-GSI had a respectable range including subjects with tendency towards cybersex addiction and severer psychopathological problems. In the BSTporn, the average performance was close to optimal but there also was substantial variance (see Table 2).

**Table 2.** Descriptive values of the BST, the BSI-GSI, and the s-IATsex

	Range	M (SD)	Skewness	Kurtosis
BST				
%setPersonPictures	25.43–70.52	48.82 (8.14)	0.227	0.398
%setPornographicPictures	29.25–73.20	51.00 (8.15)	–0.269	0.292
Deviation from set balance^1^	0.00–23.20	6.30 (5.26)	0.944	0.350
Deviation direction^2^	–41.36–48.43	2.18 (16.33)	–0.242	0.364
BSI-GSI	0.00–1.66	0.50 (0.39)	0.924	–0.052

s-IATsex				
sum score	12–44	19.86 (6.45)	1.392	2.225
subscale “loss of control/time management”	6–26	10.50 (4.7)	1.317	1.911
subscale “craving/social problems”	6–22	9.36 (3.07)	1.385	2.732

^1^ The values indicate the amount of deviation from optimal performance (i.e. working on the stimuli in each picture set equally often). Therefore higher values indicate worse multitasking performance. ^2^ Values below 0 indicate that a person worked on more “person pictures” than “pornographic pictures”. Values above 0 indicate that a person worked on more “pornographic pictures” than “person pictures”.				

The s-IATsex was positively correlated with the deviation from set balance in the BSTporn and with the BSI-GSI. However, the BSTporn scores representing the direction of deviation were not correlated with the s-IATsex. All correlations can be found in Table 3.

**Table 3.** Correlations between values of the BST, the BSI-GSI and the s-IATsex

	2	3	4	5	6	7	8
BST							
1 %setPersonPictures	–1.00**	–.035	–1.00**	.089	–.011	.035	–.070
2 %setPornographicPictures		.029	1.00**	–.097	.004	–.040	.062
3 Deviation from set balance		–	.033	.108	.286**	.193*	.343**
4 Deviation direction			–	–.092	.009	–.037	.067
5 BSI-GSI				–	.329**	.266**	.336**

s-IATsex							
6 Sum score					–	.926**	.866**
7 Subscale “loss of control/time management”						–	.614**
8 Subscale “craving/social problems”							–

* *p* ≤ .050 ** *p* ≤ .010							

**Fig. 1.**
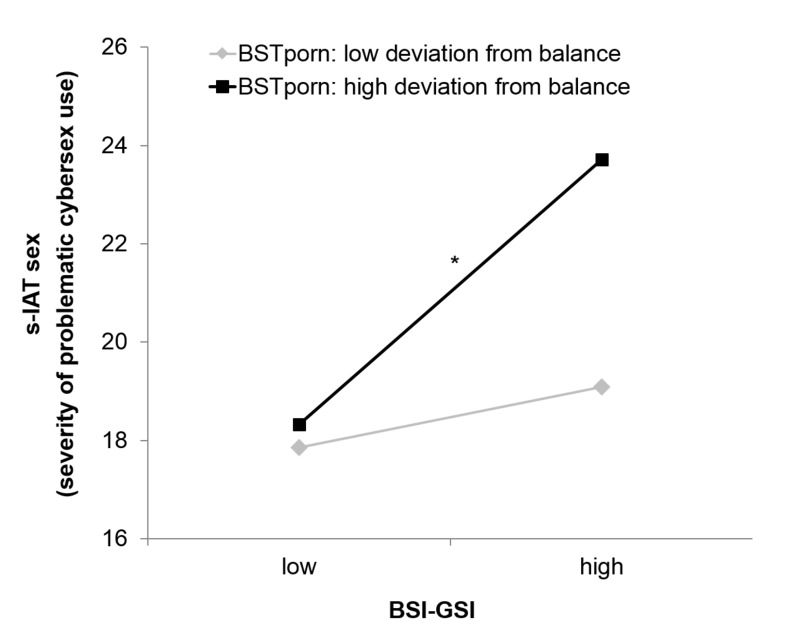
Results of the simple slope analysis of the moderated regression with s-IATsex as dependent variable and BSI-GSI and BST deviation from set balance as predictors

To test the hypothesis that particularly persons with a combination of psychopathological predisposition and reduced multitasking performance have a higher tendency towards cybersex addiction, we computed a hierarchical moderated regression analysis (Cohen et al., 2003). In the first step of the regression model, with s-IATsex sum score as dependent variable, the BSI-GSI (psychopathological predisposition) significantly explained 11% of the variance of the s-IATsex, *R*^2^ = .11, *F*(1, 102) = 12.35, *p* < .001. In the second step, the variable deviation from set balance (multitasking performance) significantly explained additional 6% of the variance of the s-IATsex, ∆*R*^2^ = .06, ∆*F*(1, 101) = 7.76, *p* = .006. In the third step, the interaction between the two predictors (BSI-GSI multiplied with deviation from set balance) significantly explained further 4% of the s-IATsex, ∆*R*^2^ = .04, ∆*F*(1, 100) = 4.88, *p* = .030. Further regression values can be found in Table 4. The interaction effect is illustrated with simple slope analysis, in Figure 1.

**Table 4.** Values of the regression analyses with s-IATsex as dependent variable

	Predictor	β	*T*	*p*	Changes in *R*^2^
Moderated regression:					
Step 1	BSI-GSI	.07	0.48	.634	.11
Step 2	Deviation from set balance	.20	2.13	.036	.06
Step 3 (interaction)	BSI-GSI × Deviation from set balance	.32	2.21	.030	.04

Curve-linear regression:					
Step 1	Deviation direction	<.01	.02	.984	<.001
Step 2	(Deviation direction)2	.33	3.52	<.001	.11

Meaning of changes in *R*^2^: In each line of the table, the changes in *R*^2^ denote how much additional variance of s-IATsex was explained by the variable when this was added as a further predictor.					

The grey line in the figure shows that persons with low deviation from set balance had low s-IATsex scores independent of whether they had high or low BSI-GSI scores. Accordingly, the slope was not significant, *t* = 0.75, *p* = .457. In contrast, the black line shows that particularly persons with high deviation from set balance, combined with high BSI-GSI scores, had significantly higher s-IATsex scores, *t* = 4.03, *p* < .001. (Please note: The points “high” and “low” represent estimated values for participants with scores one standard deviation above or below the mean of the sample. For this analysis it is not necessary to split the sample (Cohen et al., 2003).)

While the general deviation score was correlated with the s-IATsex, the variables indicating a higher occupation with one of the two sets were not. In other words, the problems users with higher s-IATsex scores had with multitasking performance were not due to an over-occupation with the pornographic pictures but also not to an over-occupation with the person pictures. So, the question remained, in which way users with high s-IATsex scores deviated from set balance.

**Fig. 2.**
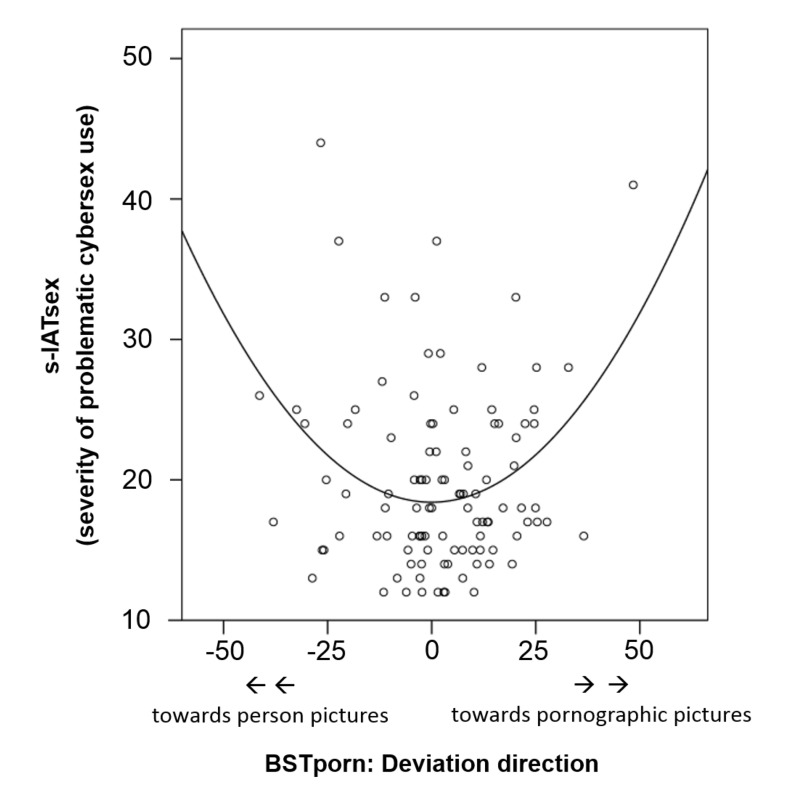
Relationship between s-IATsex and the direction of deviation from balanced working on the two task sets of the multitasking task

In an additional exploratory analysis, we tested whether the relationship between the deviation direction and the s-IATsex was not linear but u-shaped. To test this hypothesis we calculated a curve-linear regression analysis with s-IATsex as dependent variable. In the first step, deviation direction was entered as independent variable, but did not significantly explain variance of the s-IATsex, *R*^2^ < .01, *F*(1, 102) < 0.01, *p* = .930. In the second step, the squared deviation direction was entered which significantly explained 11% of the variance of the s-IATsex, ∆*R*^2^ = .11, ∆*F*(2, 101) = 12.41, *p* < .001. The u-shaped relationship is illustrated in Figure 2, further values of the regression can be found in Table 4. The estimated curve indicates that persons with high s-IATsex scores tended to work too much either on the person pictures or the pornographic pictures.

## DISCUSSION

We investigated whether a tendency towards cybersex addiction is associated with problems in exerting cognitive control over a multitasking situation that involves pornographic pictures. We used a multitasking paradigm in which the participants had the explicit goal to work to equal amounts on neutral and pornographic material. We found that participants who reported tendencies towards cybersex addiction deviated stronger from this goal.

Furthermore, as known from previous studies, the tendency towards cybersex addiction was predicted by psychopathological symptoms (see e.g., Brand et al., 2011; Brand et al., 2014; Kuss & Griffiths, 2012a; Putnam, 2000; Young, Cooper, Griffiths-Shelley, O’Mara & Buchanan, 2000). Especially persons in whom a high psychopathological predisposition and a strong deviation from the goal in the multitasking task co-occurred indicated severer symptoms of cybersex addiction.

The results are in line with the ideas by Brand et al. (2014) who pointed out that cognitive control processes, particularly executive control functions, as they are involved during multitasking, are an important component in cybersex use. On the functional side of cybersex use, executive control could be responsible for realizing goal-oriented behavior and for avoiding loss of control during cybersex use. On the dysfunctional side, problems with executive control, such as those potentially responsible for a failure to perform optimal in the multitasking task, may contribute to the symptoms of Internet addiction. In particular, problematic Internet users report that they have problems with disengaging from their preferred material, although other obligations are pending (e.g., Kuss & Griffiths, 2012a; Morahan-Martin & Schumacher, 2000; Widyanto & McMurran, 2004; Young, 1998). However, previous studies suggested that Internet addicts do not suffer from executive deficits in general (Dong, Lin, Zhou & Lu, 2013; Dong, Lu, Zhou & Zhao, 2010; Sun et al., 2009) but when they are confronted with material related to their specific addictive tendencies (Brand et al., 2014; Zhou, Yuan & Yao, 2012). Conclusions about this effect can be drawn by taking the concept of cue-reactivity (see Carter & Tiffany, 1999) into account: Excessive cybersex users may be conditioned to experience or expect reward when seeing the material and this conditioned response interferes with cognitive control processes. As a result, it may become difficult to control behavior and cognition in accordance with a previously set goal.

But which executive control function does the BSTporn demand in particular? Following our earlier work (Schiebener et al., 2014), we argue that the task should primarily load on monitoring, because it requires participants to continuously monitor the task goal (performing on equal amounts on all tasks) with respect to one’s own behavior (how often and how long the different tasks have been processed so far). Given the importance of keeping this information activated and updated continuously BSTporn performance may involve a substantial working-memory component. Working memory has been found to be interfered by the presentation of sexual stimuli (Laier, Schulte et al., 2013). In sum, the potential of sexual picture processing to interfere with working memory and executive control in multitasking situations may be seen as an important factor in loss of control as it is reported by problematic cybersex users.

Such an interference mechanism may be explained by processes taking place on the brain level. Parts of the prefrontal cortex, such as the dorsolateral prefrontal cortex, are in major control over cognitive control processes, including working memory, executive functions, and therefore also multitasking (e.g., Alvarez & Emory, 2006; Burgess, 2000; Burgess et al., 2000; Clapp, Rubens, Sabharwal & Gazzaley, 2011; Hill, Bohil, Lewis & Neider, 2013; Shallice & Burgess, 1991; Smith & Jonides, 1999; Stuss & Knight, 2013). So called fronto-striatal loops connect the prefrontal cortex with subcortical areas of the limbic system that processes emotion, motivation, and reward, particularly the amygdala and the nucleus accumbens (Alexander & Crutcher, 1990; Chudasama & Robbins, 2006; Heyder, Suchan & Daum, 2004; Hoshi, 2013). In research on substance addictions it has been shown that presenting addicted individuals addiction-cues (e.g., a picture of an alcoholic beverage) elicits strong reactions of reward processing areas but reduces prefrontal control (Bechara, 2005; Goldstein et al., 2009; see also Brand et al., 2014). In line with this view, brain imaging studies on Internet addiction also found activations of reward processing areas (e.g., nucleus accumbens; Ko et al., 2009) and changes in prefrontal activations during the presentation of addiction-specific material (see e.g., Han et al., 2011; Han, Kim, Lee, Min & Renshaw, 2010; Lorenz et al., 2013). Such a mechanism may explain the results of the current study: In persons with higher scores on the s-IATsex, the pornographic pictures may have led to activation of the reward system but reduced control of prefrontal areas which would have been important for goal-adequate performance.

While users with higher tendency towards cybersex addiction deviated more from the general goal of the multitasking task as hypothesized, they did not “get stuck” with the pornographic pictures. Instead, there was a u-shaped relationship between usage of the two sets and the tendency towards cybersex addiction. There was a small effect indicating that users with more symptoms of cybersex addiction either tended to overuse or neglect pornographic pictures.

This result may be discussed with regard to theory on approach and avoidance motivation (Elliot, 1999, 2006). The motivation to approach an event is thought to be driven by the expectation of positive implications (e.g., immediate reward), while the motivation to avoid an event is driven by the expectation of negative consequences (e.g., long term harms). Accordingly, in the literature on substance addictions (e.g., alcohol addiction) it has been pointed out that addiction cues can elicit both an inclination to approach consumption as well as an inclination to avoid consumption (Breiner, Stritzke & Lang, 1999). The final decision for approaching or avoiding consumption is said to depend on the subjective weight an addicted person currently assigns to the positive and negative consequences of consumption. Thus, one may speculate that some users with tendency towards cybersex addiction approached pornographic pictures because they lay high weight on the anticipated immediate positive effects. In contrast, others avoided pornographic pictures because they lay high weight on the anticipated negative effects. On the side of positive effects, sexual arousal can be seen as the most prominent motivator. On the side of negative effects one can assume the following motivators: Anticipation of loss of control, anticipation of unpleasant craving experiences, and fear of being convicted/negatively evaluated by the experimenter due to an overuse of the pornographic material.

Some limitations of the current study should be mentioned. First, given that the current study and the multitasking paradigm have not been designed to investigate approach and avoidance tendencies, future studies will be needed to first replicate and then better understand the observed approach vs. avoidance phenomenon. Second, the BST is a relatively new task. Although it seems to be face-valid of measuring monitoring, empirical data will be needed to verify this assumption. Third, the recruitment of the current study may have been biased because it was explicitly stated that the study is about and includes pornographic material.

## CONCLUSION

The results of the current study point towards a role of executive control functions, i.e. functions mediated by the prefrontal cortex, for the development and maintenance of problematic cybersex use (as suggested by Brand et al., 2014). Particularly a reduced ability to monitor consumption and to switch between pornographic material and other contents in a goal adequate manner may be one mechanism in the development and maintenance of cybersex addiction. This seems to be particularly the case in persons with higher psychopathological symptoms predisposing them towards developing cybersex addiction.

